# The Role of Tumor-Associated Macrophages in the Progression and Chemoresistance of Ovarian Cancer

**DOI:** 10.3390/cells9051299

**Published:** 2020-05-22

**Authors:** Marek Nowak, Magdalena Klink

**Affiliations:** 1 Department of Operative Gynecology and Gynecologic Oncology, Polish Mother’s Memorial Hospital-Research Institute, 93-338 Lodz, Poland; mrn@poczta.onet.pl; 2 Institute of Medical Biology, Polish Academy of Sciences, 93-232 Lodz, Poland

**Keywords:** ovarian cancer, tumor-associated macrophages, cytokines, chemoresistance

## Abstract

Tumor-associated macrophages (TAMs) constitute the main population of immune cells present in the ovarian tumor microenvironment. These cells are characterized by high plasticity and can be easily polarized by colony-stimulating factor-1, which is released by tumor cells, into an immunosuppressive M2-like phenotype. These cells are strongly implicated in both the progression and chemoresistance of ovarian cancer. The main pro-tumoral function of M2-like TAMs is the secretion of a variety of cytokines, chemokines, enzymes and exosomes that reach microRNAs, directly inducing the invasion potential and chemoresistance of ovarian cancer cells by triggering their pro-survival signaling pathways. The M2-like TAMs are also important players in the metastasis of ovarian cancer cells in the peritoneum through their assistance in spheroid formation and attachment of cancer cells to the metastatic area—the omentum. Moreover, TAMs interplay with other immune cells, such as lymphocytes, natural killer cells, and dendritic cells, to inhibit their responsiveness, resulting in the development of immunosuppression. The detrimental character of the M2-like type of TAMs in ovarian tumors has been confirmed by a number of studies, demonstrating the positive correlation between their high level in tumors and low overall survival of patients.

## 1. Introduction

Epithelial ovarian cancer (EOC) is a heterogeneous disease that differs in their cellular origin, pathogenesis, molecular alterations, gene expression and prognosis. Five main histopathological types of EOC have been distinguished: high-grade serous, endometrioid, clear cell, mucinous and low-grade serous. The most common type (70%) is a high-grade serous ovarian carcinoma characterized by its aggressiveness, high recurrence rate, metastasis and significant chemoresistance. In fact, 70% of EOC patients are diagnosed with the advanced stage of the disease, when cancer cells leave the primary tumor. Ovarian cancer predominantly metastasizes along the peritoneum. Distant metastatic sites mainly include the lungs, skin, pleura and lymph nodes. The traditional treatment of ovarian cancer patients consists of a combination of surgery and chemotherapy. Due to late diagnosis and resistance to drugs, the survival rate is very low, and over 75% of patients with late-stage of disease die within five years of diagnosis [1,2].

To better understand the progression, metastasis and chemoresistance of ovarian cancer, it is necessary to recognize the functioning of the ovarian tumor microenvironment and primarily the activity and properties of immune cells. Our previously published studies demonstrated that lymphocytes and macrophages are key factors involved in the interplay between the immune system and cancer cells. Moreover, neutrophils, which are innate immune cells, are also an important player in the function of the ovarian tumor microenvironment. In general, various soluble factors (e.g., cytokines, chemokines, heat shock proteins) and direct cell-to cell interactions form the active network necessary for the development of local immunosuppression, allowing cancer cells to survive, grow and develop metastatic properties [3,4,5,6,7]. This article focuses on the roles of macrophages and innate immunity cells in the progression and chemoresistance of ovarian cancer.

## 2. Tumor-Associated Macrophages in the Ovarian Cancer Microenvironment

The microenvironment of solid tumors is a complex structure consisting of a heterogeneous population of tumor cells, various resident and infiltrating host cells, secreted factors, extracellular matrix proteins, extracellular vesicles and vascular and lymphatic networks [8]. The tumor microenvironment (TME) of epithelial ovarian cancer is unique among other solid tumors since the cancer cells are easily shed from the primary tumor into the peritoneal cavity, where they create the sole microenvironment, called “malignant ascites”. Ascites-associated ovarian cancer cells exist as single floating cells or more frequently as multicellular spheroids. Moreover, malignant ascites also include immune cells, fibroblasts, adipocytes, mesothelial cells, extracellular microvesicles, cytokines, growth factors and lipid mediators. Except for the very early clinical stage of the disease (IA by FIGO), with disease progression, the ovarian tumor environment usually includes the primary tumor, metastases and malignant ascites as well [9,10].

Tumor progression is dependent not only on the characteristics of malignant cells but also on the behavior of the whole TME. The population of immune cells is an important player in tumor progression, including the process of metastasis. In the primary ovarian tumor and in ascites, the major population of immune cells is composed of macrophages. They originate from two sources: (1) tissue-resident macrophages, which are derived from the embryonic yolk sac and (2) infiltrating macrophages recruited from bone marrow-derived monocytes. In the TME, they are converted into tumor-associated macrophages (TAMs). TAMs are highly plastic cells, and, depending on the stimuli, they can exhibit two main phenotypes: anti-tumorigenic M1-like and pro-tumorigenic M2-like. However, in the ovarian cancer microenvironment, TAMs generally exhibit the M2-like phenotype, with high expression of scavenger receptor class B (CD163), mannose receptor (MR, CD204) that serves as markers and immunosuppressive factors, including interleukin-10 (IL-10), as well as chemokines CCL18 and CCL22. The TAMs isolated from ascites of ovarian cancer patients expressed also membrane form of IL-18. Nevertheless, mixed-polarization phenotype expressing CD163 and IL-10 (M2) are also observed simultaneously with co-stimulatory molecule (B7.2, CD86) and tumor necrosis factor α (TNF-α) (M1). In ascites, the population of M1-like phenotype expressing interferon γ (IFN-γ) and IL-12 can also be found. It has been recognized that M2 TAMs strongly support ovarian cancer growth, metastasis and resistance to therapy [10,11,12,13,14]. Since, M2-like pro-tumoral TAMs are preferentially involved in ovarian cancer progression, this review focuses especially on their activity.

**Figure 1:**
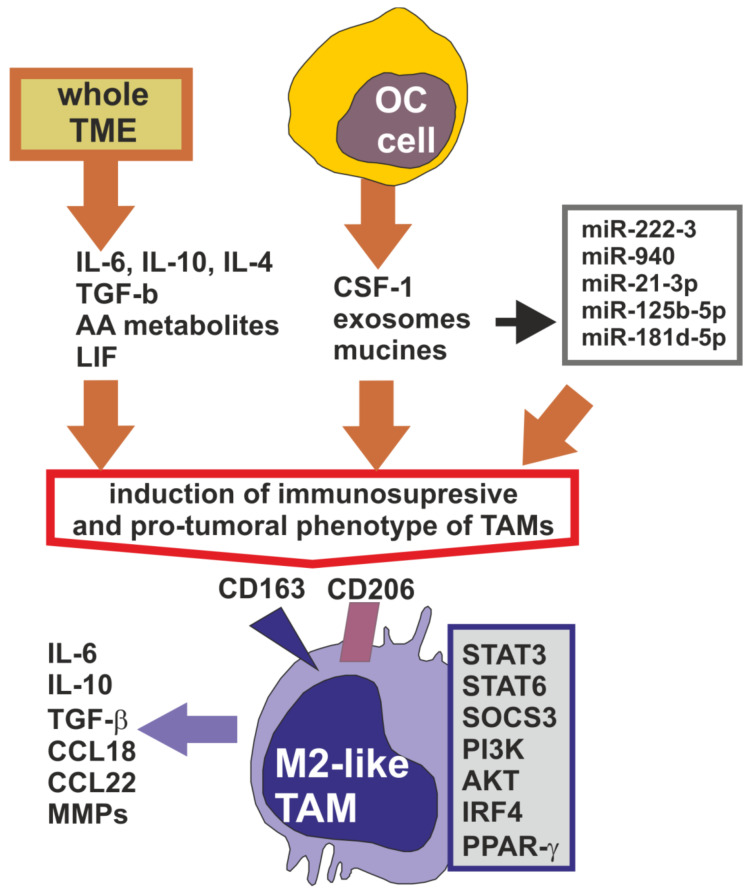
Induction of M2-like TAMs in ovarian cancer tumor microenvironment. The soluble factors (cytokines, chemokines, AA metabolites, mucines and exosomes reach in microRNAs) released by ovarian cancer (OC) cells and other cells of tumor microenvironment (TME) polarize TAMs into immunosuppressive M2-like phenotype. M2-like TAMs characterize with surface expression of CD163 and CD206 receptors, overexpression of various intracellular signaling proteins and production of immunosuppressive and pro-angiogenic and pro-invasion factors.

The polarization of TAMs is strongly dependent on various soluble and insoluble factors present in the ovarian tumor microenvironment, including both the peritoneal and primary site (Figure 1).

It is believed that the entire TME can induce TAMs to display an immunosuppressive and pro-tumoral phenotype, facilitating cancer growth and progression. However, the main players in TAMs education are the cancer cells themselves [9,15]. There are a number of well-known soluble factors, including IL-6, IL-10, IL-4, IL-13, leukemia inhibitory factor (LIF), transforming growth factor β (TGF-β) and arachidonic acid metabolites, which strongly polarize TAMs into M2-like phenotype. The pro-tumoral characteristic of TAMs is also initiated by macrophage colony-stimulating factor, also known as colony-stimulated factor 1 (CSF-1), which is considered to be critical in the induction of the M2-like phenotype of cells. The main source of CSF-1 in the TME are cancer cells [9,11,13,16].

Moreover, Allavena et al. [17] demonstrated that mucins of ovarian cancer cells activate TAMs’ immunosuppressive profile, reflected by high IL-10 production. Another TME factor that induces the M2-like phenotype is ovarian cancer-derived exosomes that are rich in a variety of microRNA (miR). It was found that miR-222-3 upregulates and activates signal transducer and activator of transcription 3 (STAT3) and suppressor of cytokine signaling 3 (SOCS3) proteins in macrophages, resulting in their polarization into immunosuppressive and pro-tumoral function [18]. One more report demonstrated that miR-940 in exosomes secreted from ovarian cancer cells and expressed in ascites of EOC patients stimulate TAMs to develop a M2-like phenotype [19]. The significant increase in the expression of CD163 and CD206 markers in macrophages was also observed after their in vitro culture with exosomes containing miR-21-3p, miR-125 b-5p and miR-181 d-5p [20]. The pro-tumoral and immunosuppressive character of M2-like TAMs is related to the overexpression and/or overactivation of selected signaling proteins, including STAT3, STAT6, phosphoinositide 3-kinases (PI3K), serine/threonine-specific protein kinase (AKT), interferon regulatory factor 4 (IRF4) and peroxisome proliferator-activated receptor gamma (PPAR-γ) [21,22].

## 3. Tumor-Associated Macrophages Facilitate Ovarian Cancer Progression

**Figure 2:**
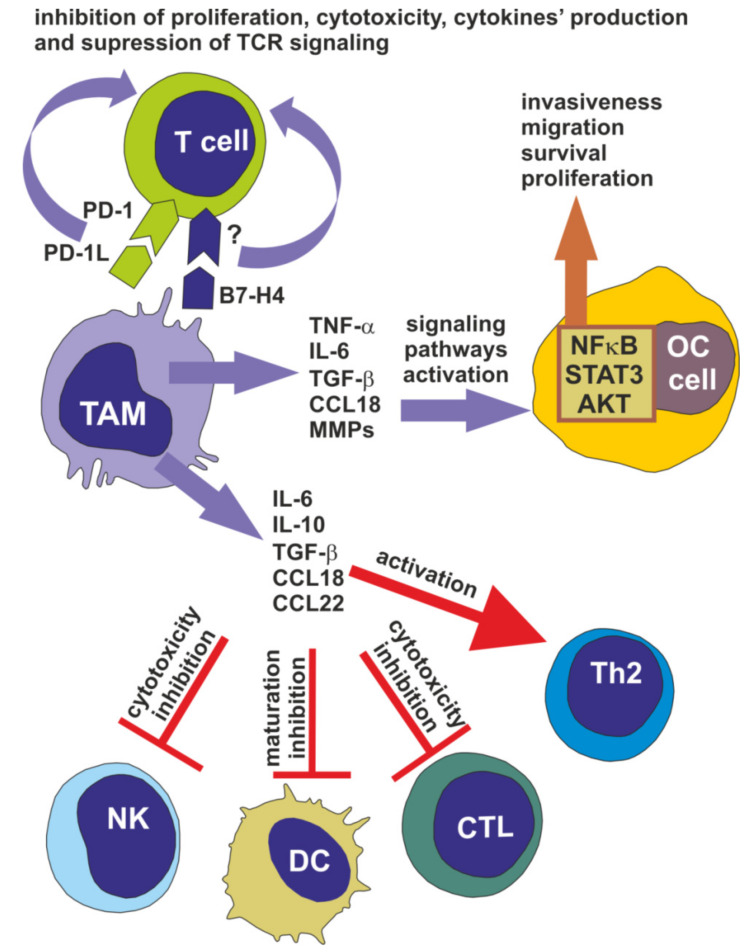
Involvement of M2-like TAMs in the progression of ovarian cancer. TAMs release a variety of soluble factors responsible for the activation of various signaling pathways in ovarian cancer (OC) cells, promoting their survival, proliferation, migration and invasiveness. Cytokines released by TAMs impair the activity of immune cells, including dendritic cells (DC), natural killer (NK) cells, and cytotoxic T cells (CTL), as well as induce the activity of Th2 cells. Direct interaction of TAMs and T cells via PD-L1 and B7-H4 inhibits the function of T cells, resulting in the immune escape of OC cells.

TAMs facilitate the progression of ovarian cancer at many stages of the disease development, including immune escape of tumor cells, cancer cell invasion, migration and metastasis and the angiogenesis process (Figure 2).

The important mechanism by which TAMs induce the invasiveness of ovarian cancer cells is via the augmentation of nuclear factor κB (NFκB) activity. Many reports from in vitro studies on various cell lines clearly indicate that TNF-α is a key player in NFκB upregulation and activation in cancer cells and TAMs are one of sources of this cytokines in ovarian TME [23,24,25]. Although, TNF-α is generally secreted by classical M1 macrophages, M2-like TAMs (classified also as M2d phenotype) express CD163 marker and produce TNF-α together with IL-10 and TGF-β [22]. In addition, as was mentioned above (Heading 2) M1-like macrophages are also present in ovarian cancer microenvironment. Moreover, macrophages using TNF-α significantly upregulate the PI3K and Akt signaling proteins to promote the migration and invasion abilities of ovarian cancer cell lines, in vitro [26]. Other cytokines secreted by TAMs that induce the invasive potential of ovarian cancer cells are IL-6 and TGF-β. IL-6 leads to activation of the STAT3 pathway [27], which is required for ovarian cancer cell migration, motility, survival and proliferation. TGF-β increases the invasiveness of ovarian cancer cell lines through the induction of various metalloproteinases’ (MMPs) production and activation, in vitro [28]. What is more, the CCL18 chemokine, the level of which is markedly increased in the ascites of advanced-stage patients, promotes cancer cell migration [29]. The in vitro studies on cell lines and cells isolated from tumor tissue of patients demonstrated that ovarian cancer cells secrete migratory inhibitory factor and extracellular matrix metalloprotease inhibitory factor, which in turn induce the production and release of MMPs by TAMs. MMPs are known factors that promote the invasiveness of cancer cells, among others, through remodeling of the extracellular matrix and induction of the angiogenesis process [22,23,30]. The ability of M2-like TAMs to promote ovarian cancer cell invasion is also related to scavenger receptor (SR) expressed on their surface, as reported in studies using both cell lines and mice knockdown models. However, the ligand for SR and the exact mechanism of its impact on cancer cells is unknown [15,31]. Another study demonstrated that high expression of insulin growth factor 1 (IGF-1) by mouse TAMs induces migration of mouse epithelial ovarian cell lines [32].

The M2-like TAMs participate in ovarian cancer progression through their significant immunosuppressive impact on immune cells in the TME, which allow for the evasion of cancer cells. This conclusion is based on primarily on in vitro studies involving both ovarian cancer cell lines and cancer cells isolated from patients’ tumor tissue. TAMs secrete IL-10, IL-6, TGF-β, CCL18, and CCL22, which attract regulatory T cells and promote differentiation of T cells towards the Th2 phenotype. Moreover, IL-10 and TGF-β inhibit the cytotoxic activity of natural killer cells and cytotoxic T lymphocytes. In addition, IL-10 blocks maturation of dendritic cells. CCL18 promotes T cells’ anergy and unresponsiveness [33,34]. The immunosuppressive function of M2-like TAMs is strongly associated with overactivation of STAT3 protein, which in turn upregulates IL-10 and IL-6 production [35].

Another mechanism by which M2-like TAMs regulate T cell activity is by their influence on checkpoint programmed cell death protein 1 (PD-1). The interaction of PD-1 with its ligand PD-L1 on macrophages induces T cells to become unresponsive, manifesting in inhibition of their proliferation, cytotoxicity, the production of cytokines and the suppression of TCR and/or costimulatory signaling, finally resulting in the blocking of tumor-specific T cell responses. The main signaling pathways inhibited via triggered PD-1 are the PI3K/AKT and Ras-MEK-ERK pathways [36,37,38]. The PD-L1-positive TAMs are common in both primary and metastatic high-grade serous ovarian carcinoma and are correlated with the acute aggressiveness of this type of EOC [39]. Interestingly, the PD-L1-positive TAMs isolated from tumor tissue effectively induce apoptosis of autologous blood CD8^+^ T cells [40]. It was also reported that more than 70% of TAMs of ovarian cancer patients are characterized by the expression of B7-H4, a coinhibitory molecule [41]. B7-H4 is a transmembrane protein belonging to the B7 family of costimulatory proteins. However, its ligand on T cells is presently unknown. Nevertheless, B7-H4 binds the putative receptor on activated CD4^+^ and CD8^+^ T cells, causing a decrease in their proliferation and reduction in IL-2 production, as was reviewed by Smith et al. [42]. The in vitro studies on TAMs isolated from ovarian tumor tissue and ascites of patients indicates that the expression of B7-H4 on them is induced by IL-6 and IL-10 [34].

The most common route of metastasis of ovarian cancer cells is called the transcoelomic route, and it relies on the detachment of cancer cells from the primary tumor and their movement through the peritoneal fluid to the omentum, parietal and visceral peritoneum, as well as the direct extension of tumor lesions to adjacent organs. The additional route is via lymphatic vessels to pelvic and paraaortic lymph nodes. The peritoneal dissemination of metastases is relatively uninhibited, since lack of an anatomical barrier around the primary tumor allows for the shedding of cancer cells. However, peritoneal and then ascitic fluid are necessary for successful transcoelomic metastasis. The etiology of ascites formation is not well-understood, but the most important prominent inducing factor seems to be vascular endothelial growth factor (VEGF). Malignant cells survive in the peritoneal cavity as free-floating single cells, or they aggregate as multicellular spheroids. The cancer cells have to survive in the anchorage-independent condition and thus should be resistant to anoikis (programmed cell death due to detachment of cells from the extracellular matrix). The attachment of spheroids to peritoneal organs involves interaction between the cancer cells and the mesothelium. It should be underlined that the classic patterns of metastasis via the hematogenous route also occur in ovarian cancer, but it is not the dominant way, and it is rather responsible for distant metastases [43,44,45].

The peritoneal cavity is a large area lacking blood and lymphatic vessels and matrix which could support ovarian cancer cells from anoikis. As to the question of how ovarian cancer cells can survive in the peritoneum, the answer is TAMs. In the malignant ascites/intraperitoneal milieu, M2-like TAMs play a pivotal role in the transcoelomic dissemination of ovarian cancer cells, their survival in ascitic fluid and the formation of spheroids. What is more, a great number of spheroids in ovarian cancer patients are heterogeneous, consisting of TAMs-OC cells [46]. The epidermal growth factor (EGF) released by TAMs increases the expression of ICAM-1 in cancer cells and upregulates the αMβ2 integrin in macrophages, facilitating both cells’ interactions. In turn, the interaction between TAMs and OC cells enhance EGF production. The effect of EGF on ICAM-1 occurs through the induction of VEGF-C expression, which in turn activates VEGFR3 signaling and induces integrin/ICAM-1 expression [45,47]. Moreover, IL-6 and IL-10 released by blood monocytes-derived TAMs activate the pro-survival STAT3 signaling in ovarian cancer cell lines and cancer cells isolated from patients’ ascites, keeping them alive [48].

The omentum, the most common site of ovarian cancer cell metastasis, is reached by macrophages. They are located in areas called milky spots, which are preferentially colonized with ovarian malignant cells. Lack of a basement membrane and few mesothelial cells on milky spot surfaces facilitate the attachment of cancer cells [43,49]. As was detailed described in other review article [43,45,46] the secreted by M2-like omental macrophages, proangiogenic and extracellular matrix protein remodeling factors such as MMP9, VEGF-C, TGF-β are key players in the successful implantation of spheroids. Attached cancer cells proliferate and invade the metastatic area.

## 4. Tumor-Associated Macrophages Participate in Ovarian Cancer Chemoresistance

The standard therapy for ovarian cancer patients consists of cytoreductive surgery (total abdominal hysterectomy, bilateral salpingo-oophorectomy with tumorectomy, omentectomy, appendectomy, metastasectomy—the goal is to leave no residual macroscopic disease), followed by the administration of chemotherapeutics (platinium-based compounds with taxans). Although initial response is promising, recurrence is frequently observed, usually between 6 and 24 month after treatment, and many patients die within 5 years. Most relapsed patients acquire platinium resistance due to repeated chemotherapy cycles. On the other hand, the primary platinium-resistant ovarian cancer cells are also present in tumor tissue. Therefore, resistance to chemotherapy, both intrinsic or acquired, is a common problem in the treatment of ovarian cancer patients [50,51].

Although the mechanisms leading to ovarian cancer chemoresistance are not fully clear, some factors have been proposed. Tumor microenvironment is a potential player in recurrence and resistance to chemotherapy. The multifaceted cooperation of ovarian cancer cells and TAMs in TME is suggested as a one of the causes of treatment failure. This proposal is based on many in vitro and in vivo studies that have demonstrated that depletion of TAMs attenuates cancer chemoresistance. Importantly, involvement of TAMs in chemoresistance is mainly recognized in breast cancer. However, in the case of ovarian tumors, their involvement has also been found, although the number of published studies asserting this are rather limited [22]. According to the available literature, we can summarize that the main mechanisms of TAMs-related ovarian cancer chemoresistance include: (i) the pro-tumoral polarization of macrophages; (ii) the impact of macrophages on the pro-survival signaling pathways; and (iii) upregulation of multidrug resistant genes in cancer cells by macrophages. Below, we present a review of selected studies supporting the role of TAMs in ovarian cancer chemoresistance.

A study described by Dijkgraaf et al. [52] proposed an indirect mechanism of ovarian tumor chemoresistance through the platinium-induced promotion of M2-like macrophages. They found that various ovarian cancer cell lines treated with cisplatin or carboplatin induced differentiation of macrophages into an M2-like phenotype characterized by elevated production of IL-10 and enhanced activation of STAT3 signaling factor. The IL-6 and prostaglandin E_2_ realized by platinium-treated cancer cells were responsible for polarization of macrophages. The immunosuppressive M-2- like TAMs are implicated in cancer progression, as was discussed above.

Although the M1-like macrophages are believed to be anti-tumoral and pro-inflammatory, the platinium compounds can change their beneficial activity. Studies by Liu et al. [53] clearly demonstrated that cisplatin modulates M1 macrophages, inducing their ability to stimulate the migratory property in SK-OV-3 and A2780 ovarian cancer cell lines. Although, in general, the M1 phenotype was not changed due to cisplatin treatment, this drug enhanced IL-1β and CCL20 secretion. The CCL20 chemokine was responsible for the induction of cancer cells’ migration.

An important activity of macrophages is the secretion of miR-loaded exosomes. The exosomes secreted by TAMs (M2-like phenotype) are effectively internalized by ovarian cancer cells (A2780, SK-OV-3), conferring their drug resistance. Detailed analysis has revealed that miR-223 present in exosomes activated the PI3K/Akt pathway in cancer cells. This pathway promotes cell survival and inhibits cell apoptosis. These results were also confirmed in an in vivo model of xenograft tumor. The tumors of mice injected with TAMs exosomes or miR-223 expressed activated AKT protein [54].

Another study examined the determination of genes related to drug resistance in cisplatin-sensitive A2780 and cisplatin-resistant A2780cis ovarian cancer cell lines cocultured with M1 and M2 macrophages. The data revealed that macrophages, independent of phenotype, induced expression of the *ABCG2* gene, which encodes protein involved in drug resistance in A2780 cells but not A2780cis cells. Additionally, ovarian cancer cells both drug-sensitive and -resistant polarize macrophages toward M2-like phenotype [55].

## 5. The Prognostic Significance of Tumor-Associated Macrophages in Ovarian Cancer

The phenotype of TAMs infiltrating ovarian cancer tissue was evaluated as a prognostic factor. Below, we present examples of published data that, without a doubt, indicate that M2-like phenotype is an indicator of patients’ poor prognosis. A study by Yafei et al. [56] described the prognostic significance of CD68^+^ and CD163^+^ positive macrophages in a group of 42 ovarian cancer patients at all stages of disease. Immunohistochemical analysis demonstrated that the high proportion of CD163^+^ (M2 phenotype) in whole CD68^+^ macrophages was the predictive factor of poor prognosis. Another study enrolled 108 patients with advanced stage ovarian cancer, showing that the progression-free survival (PFS) and overall survival (OS) rates were significantly higher in the group with low expression of CD163 (immunostained specimens) in comparison with the high-CD163 expression group [57]. In a meta-analysis performed by Yuan et al. [58] on 794 ovarian cancer patients to determine the correlation between TAMs phenotype and clinical outcomes, infiltration of tumor tissue with CD163^+^ TAMs was associated with poor prognosis, while a high M1-to-M2 macrophage ratio predicted better prognosis for both OS and PFS. Another study on a group of 112 patients (FIGO I-IV) also clearly indicated that a high M1-to-M2 ratio of TAMs in tumor specimens was correlated with extended survival [59]. A study with a cohort of 199 high-grade serous ovarian cancer patients found that a high M2-to-M1 ratio was associated with a decrease in PFS and poor OS [60]. A similar observation was reported by Ciucci et al. [61]. They tested 25 patients with low-grade serous carcinoma (LGSOC; better prognosis) and 55 patients with high-grade serous carcinoma (HGSOC; poor prognosis). The results showed that LGSOC patients exhibited lower levels of total (CD68^+^) as well M2-like (CD163^+^) TAMs. Vankerckhoven et al. [62] evaluated the presence of M1 and M2 TAMs in the tissue samples of primary tumors from 24 patients with ovarian cancer, mostly in advanced stage. The authors noted that low-grade ovarian cancer showed more M1 TAMs, and less M2 TAMs compared to high-grade ovarian cancer. Liu et al. [63] searched publicly available databases and performed an analysis of 13 independent studies on 2218 patients with HGSOC. The obtained data demonstrated that a high proportion of M1 phenotype of TAMs was associated with favorable OS. The present study showing the link between M2-like TAMs and unfavorable patient survival can confirm the data presented above, with considerations about their implication in cancer progression.

## 6. TAMs as Therapeutic Target in the Treatment of Ovarian Cancer

Recognition of TAMs’ involvement in tumor progression and chemoresistance has provided opportunity to develop the therapy for ovarian cancer. Three main, anti-TAMs strategies has been successfully developed and are used in various clinical trials. One strategy concerns blocking of macrophages migration and recruitment, second is based on re-polarization of macrophages from M2 to M1 phenotype, and the third is based on blocking immune checkpoint (PD-L/PD-L1). There are a number of excellent review papers describing in details pre-clinical and clinical studies of all these anti-TAMs therapies in ovarian cancer [21,22,64,65,66]. Therefore, on the basis of this vast knowledge, in this article we only summarized current achievements in this field.

The CSF-1 is a key factor for TAMs polarization into M2-like phenotype. Several inhibitors (small molecules), as well as antibodies blocking CSF-1 receptor (CSF-1R), expressed on TAMs, were developed. In mouse ovarian tumor models and in ovarian cancer patients it was shown that targeting CSF-1R reduces the infiltration of macrophages into tumor tissue and improves patients’ response to standard treatment. Moreover, blocking the CSF-1R re-polarize M2-like TAMs to M1 phenotype in tumor tissue. However, it should be emphasized that anti CSF-1R agents are used in the combinations with other compounds and molecules, such as taxanes or anti-PD-1 antibodies [21,64,66]. Repolarization (in vitro) of M2-like TAMs isolated from ascites of ovarian cancer patients into M1-like phenotype was also showed after TLR4 activation with LPS. The M1-like TAMs were able to induce NK cells activation thus macrophages lost their immunosuppressive character [14]. The expression of PD-L1 on TAMs is one of macrophages’ strategy to induce the immunosuppression in TME. The blocking of PD-1/PD-L1 interaction by antibodies directed against PD-L1 molecule seems to be promising in advanced, platinum resistant ovarian cancer patients. The clinical trials that target PD-L1 involves also anti-VEGF antibody or inhibitor poly ADP ribose polymerase or inhibitor of VEGF receptor tyrosine kinases [22,64,66]. One of the factors, that regulates the influx of circulating monocytes into tumor tissue, is CCL2 chemokine also known as monocyte chemoattractant protein 1 (MCP-1). Thus CCL2/CCLR axis is attractive target for ovarian cancer therapy. Antibodies against CCL2 are currently tested in clinical trials in patients with advanced stage of disease. However, similarly to other anti-TAMs strategies, the anti-CCL2 antibodies are used in combination with chemotherapeutics including paclitaxel, gemcitabine, carboplatin or docetaxel. The CCL2R antagonist are still under development but so far it has shown to decrease the number of bone-marrow derived macrophages in tissue in the mouse model [22,64,65].

Despite the promising results of using anti-TAMs strategies in clinical trials, this kind of ovarian cancer treatment is still narrow. Firstly, anti-TAMs therapy needs to be combined with other drugs. Secondly, macrophages are very plastic cells that can change their activity very easily in response to changes in TME. Thirdly, the interactions of TAMs with ovarian cancer cells or with other immune cells are very complex what significantly limits effectiveness of blocking one chosen cells’ activity.

## 7. Summary

The ovarian tumor microenvironment is a unique and complex structure consisting of primary tumor and malignant ascites. In both components, the major population of immune cells is comprised of TAMs. They are highly plastic cells and easily respond to environmental stimuli. Ovarian tumor cells, mainly via CSF-1, induce TAMs to exert pro-tumoral activity. TAMs, especially those of M2-like phenotype, are the source of various cytokines, chemokines, enzymes and exosomes that increase the invasion potential of tumor cells and promote immunosuppression, allowing cancer cells to survive, proliferate and metastasize. Moreover, chemoresistance of ovarian cancer partially depends on the multifaceted cooperation of macrophages with tumor cells. The main factors affecting the biological activity of cancer cells are IL-10, IL-6, TGF-β, VEGF, CCL18, CCL22, and microRNA. Nevertheless, direct interaction of TAMs with T cells via PD-L1/PD-1 is a main cause of the downregulation of anti-tumor activity among T cells. The negative role of TAMs in ovarian cancer are confirmed by clinical examination showing that high infiltration of tumor with M2-like phenotype correlates with poor prognosis and unfavorable OS.
